# Children who were vaccinated, breast fed and from low parity mothers live longer: A community based case-control study in Jimma, Ethiopia

**DOI:** 10.1186/1471-2458-11-197

**Published:** 2011-03-31

**Authors:** Belaineh Girma, Yemane Berhane

**Affiliations:** 1 Department of Public Health and Social Science, Addis Continental Institute of Public Health, P.o.box 26751/1000. Addis Ababa, Ethiopia; 2 Department of Epidemiology & Biostatistics, Addis Continental Institute of Public Health, P.o.box 26751/1000. Addis Ababa, Ethiopia

## Abstract

**Background:**

Improving child survival through various health interventions has been one of the main preoccupations of public health programs in developing nations. However, efforts to understand the child death determinants and determine whether the health interventions are really contributing to the reduction of mortality were not satisfactory. The purpose of this study is to identify determinants and causes of child mortality.

**Methods:**

The study was conducted in the town of Jimma, Ethiopia, using a case control study design. Cases were identified through enumeration of all children and deaths prior to interview of the study subjects. Controls were under five children of the same age (+/-2 months) residing in the nearest household. Data was entered into EPI -info 6.4 software and analyzed using SPSS.

**Results:**

Seventy four cases and 222 controls were included in the study. The study found that children who never breast fed [OR = 13.74, 95%CI (3.34, 56.42]] and children with mothers having more than five children [OR = 3.34, 95%CI (1.27, 8.76)] were more likely to die than their counterparts. Vaccination reduced the risk of death [OR=.26, 95%CI (0.10, 0.67) significantly. Pneumonia was the most common immediate cause of death [29.7% (95% CI (19.66, 41.48)] followed by acute diarrhea and malaria each contributing for 23% [95%CI (13.99, 34.21)] of deaths.

**Conclusion:**

Immunization, breastfeeding and low parity mothers were independently found to be protective from childhood death. Strengthening the child survival initiatives, namely universal child immunization, family planning and breast feeding -- is strongly recommended.

## Background

The estimate for global child deaths in 2000 was 10·8 million. About 41% of child deaths occur in sub-Saharan Africa Worldwide, half deaths in children younger than five years occur in only six countries and 90% in 42 [1].

Substantial reductions in child mortality occurred in low-income and middle-income countries in the late 20^**th **^century [1]. Rates of decline in global child mortality peaked in about 1980. In 1990-2001, the number of child deaths fell by 1·1% every year compared with 2·5% per year during 1960-90. Decrease in rate of decline has occurred in high-rate regions [1].

The conceptual framework for study of determinants of child survival includes proximate and distal (socioeconomic) determinants. This approach to the study of child survival is based on several premises [2]. Many studies have shown that poor income, maternal education and illiterate fathers are risk factors for infant neonatal and child deaths [3-9].

Studies identified short birth interval, high birth order and young or older age at maternity as hazards of death of infants and children. Other studies also revealed no significant effects for some of the factors [8,10,11].

Unhygienic and unsafe environments place children at higher risk of death [1]. Type of roof, floor of household latrine facility and soap use are associated with infant mortality [10]

According to literatures infants aged 0-5 months who are not breastfed have seven-fold and five-fold increased risks of death from diarrhea and pneumonia respectively, and breast feeding predicts child mortality [1,12].

Child survival interventions are not reaching the children who need them most. The inequities between poor and well to do are accompanied by reduction in access to preventive and curative service [2]. Measles vaccine was received by two-thirds of children younger than 5 years, and all other interventions had coverage of less than 60%. Among Sub-Saharan African countries with endemic malaria, results of surveys showed that a median of less than 2% of children slept under an insecticide-treated net[13].

In countries with poor data on mortality, vital registration are weak and the proportion of people who died while under medical care is low. In such situations a verbal autopsy, a tool which uses ascertainment of causes of deaths from data obtained from relatives or associates of the deceased through retrospective questioning in surveys or in demographic surveillance systems seems an attractive options [14,15].

The Ethiopian situation is also similar to that of Sub-Saharan Africa, characterized by a high child mortality rate. Ethiopia ranks 6^th ^in the world by total number of deaths of under five children. Under five mortality in Ethiopia is high (123 per 1000 live births) which declined from 166/1000 live births Data from the Ethiopian Demographic Health Survey showed that mortality has declined in Ethiopia over the past 15 years and that this decline is more pronounced over the last 10 years. There is a declining trend infant and child mortality rate consistently over the past 15 years [4,16,17]..

Ethiopia is a country where low standard of living, poor environmental conditions and underdeveloped social services prevails. It is also characterized by absence of vital event registration system and inaccessibility of health facilities which forces deaths to occur at home. In addition to this, the studies were conducted a decade back and current public health challenges are not comprehensively addressed in the literature.

Understanding the epidemiological profile of child death and the capabilities of health systems and approaches in dealing with childhood health problems in a local context is essential to come up with public health interventions that are relevant. Development of these interventions also requires an understanding of the determinants of child survival.

Thus the purpose of this study is to identify determinants and causes of child mortality that would help in planning and implementing interventions to reduce under five deaths.

## Methods

### Study setting

The study area is Jimma town, located 335 km from Addis Ababa. The projected population of Jimma in 2004 was about 120,000 [18]. The town had 21 kebeles/the smallest administrative unit. The majority of the population is Oromo by ethnicity. The study was conducted from October 1,2004 to November 8, 2004.

### Study Design

The study utilized a case control design.

### Study Population

The source of the population was all under five children in Jimma who died in the previous one year.

Study population included all cases identified and their controls.

Cases were all identified deaths among under five children in one year prior to data collection. Controls were living under five children of the same age (+/-2 months) living in the nearest household with the case at the time of the survey.

The sample size was calculated using STATCALC program of EPI 6.04 Software. The minimum number of cases and controls required to achieve a 95% confidence level and power of 80% were calculated. Three controls were taken for each case.

A baseline survey was conducted to enumerate under five children and identify deaths during a one year period. For each identified case 3 controls were chosen from the next household. If two or more eligible controls were found in the same household the one closest to the age of the case was selected.

### Data collection

The questionnaire (Additional file 1) had five parts. The first part was used to list all under five deaths that occurred in one year period prior to data collection. The second part consisted sections on socio demographic and proximate factors of under five deaths. The third part had morbidity pattern questions for controls. The fourth part was a verbal autopsy tool for neonatal deaths. The final part was a tool to identify causes of death in the post neonatal period. The fourth and fifth were modified from WHO standard questionnaire[16]. The modification was done to make the instrument relevant and applicable in the local context. The questionnaire was initially prepared in English, and translated into Amharic. The consistency was checked by back translation to English. The instrument was pilot tested.

Interviews had taken place with mothers or guardians of the selected child. The supervisors rechecked 5% of the study population and reviewed the completed questionnaire daily.

The main outcome variable was under five deaths. Explanatory variables were: socio-demographic variables (age of the child, sex, religion, ethnicity, and age of father, age of mother, educational status of father and mother and income) and proximate determinants of child survival which include reproductive health factors (age at first pregnancy, age at birth of the child, birth order, birth interval, antenatal care attendance, delivery attendance and family planning), environmental factors (presence of latrine, water source, and construction materials of the house), nutritional factors (breastfeeding pattern and practice) child factors (immunization, birth weight) and causes of death of a deceased child.

### Data Analysis

The data were edited then entered into EPI info 6.04 and SPSS version 11 software was used for data entry and analysis. Frequencies and cross tabulations were used to check consistency. Composite scales were constructed to represent a single construct.

***Wealth score ***was a composite scale used to assess the economic status of the family. This index is believed to include elements of deprivation and income which gives reliable information about the level of poverty [19]. Items included were income, presence of radio and television, and presence of latrine and water source. The sum was computed and those who scored above the median value were classified as having satisfied basic needs. Eight ***knowledge ***items were used to assess the *knowledge *of care takers. The sum was computed and those who scored more than the median value were labeled as having good knowledge.

Child health practices were assessed using four items, asking participants whether the care takers use preventive or curative service for the most common childhood problems. From these items those with scores greater than the median value were categorized as having good child health care practices.

The data were summarized using proportion, means and medians. Bivariate analysis was done to assess the level of association between independent variables and outcome variable. Odds ratios were computed to determine the strength of association. A 95% Confidence interval was used to estimate the level of significance. logistic regression was used to control the effect of confounding variables. Variables with a significant level of significance and from bivariate analysis were included in a backward stepwise logistic regression procedure. The variables included were: ethnicity, religion, fathers' age, floor materials of the house, parity, ever breast fed, vaccination status, and wealth index.

The cause of death was determined using expert algorithm [20]. The expert algorithms were constructed to incorporate major diseases that are prevalent in the country. The causes of death were determined by using algorithms from other studies. The algorithm to classify HIV/AIDS cases were constructed using WHO clinical case definition for pediatric AIDS[21]. The analysis tried to assess the contribution of HIV/AIDS and malnutrition to under five deaths as underlying cause of death. Multiple causes of death were entertained.

### Ethical considerations

Ethical clearance was obtained from the Ethical Review Board of the Faculty of Medicine, Addis Ababa University. Informed consent was obtained from each participant after informing them about their right to withdraw from the process and refuse participation. They were also informed about the confidentiality of the information they provided. The interview was conducted after the mourning period of 40 days for all cases. If children found ill during the survey, study personnel advised their care takers to take them to the nearest health facility.

## Results

The total number of under five children from the baseline survey was 6711. Males account for 52% of the total population and females 48%. The total number of deaths was 74.

**Figure 1:**
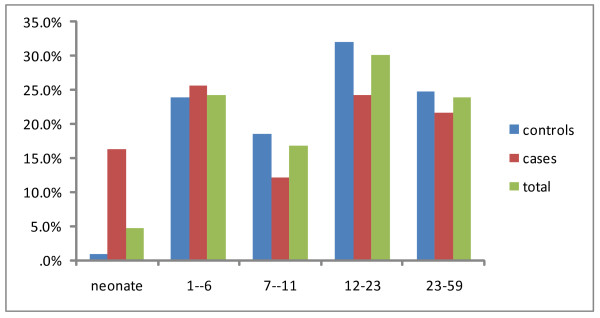
**Age distribution of the study participants disaggregated by outcome status**. Jimma town, Southwest Ethiopia, October-November 2004

The total number of cases and controls were 74 and 222 respectively. Among the study population males constitute 170 (57.4%)(Table 1)Most cases were in the age group of 1-6 months(figure 1). Most of the respondents were mothers (92.9%). The lowest and highest reported income were 15 and 2620 birr respectively. The median monthly income was 200 birr.

**Table 1:** Socio demographic characteristics of the study participants and their care takers, Jimma town, Southwest Ethiopia, October-November 2004

*Variable*	*Category*	*Number*	*%*
Age of a child (months)	Neonate	14	4.7
	1-6	72	24.3
	7-11	50	16.9
	12-23	89	30.1
	23-59	71	24.0
Sex	male	170	57.4
	female	126	42.6
Religion	Orthodox	136	45.9
	Protestant	33	11.1
	Muslim	127	42.9
Ethnicity	Oromo	146	49.3
	Amhara	49	16.6
	Garage	19	6.4
	Dawro	34	11.5
	Yem	23	7.8
	Others	25	8.4
Wealth index	Poor	227	76.7
	Satisfied basic needs	69	23.3
level of education(mother)	illiterate	37	12.9
	primary	102	35.5
	secondary and Tertiary	148	51.6
Level of education(father) (father	illiterate	13	5.3
	primary	72	29.3
	Second and Tertiary	161	65.4
Age category(father)	15-29	77	26.0
	30-49	197	66.6
	50+	22	7.4
Age category (mother)	15-19	26	9.0
	20-29	193	66.8
	30 +	70	24.2

Reduced mortality was observed among female compared to males, but the association was not significant [*(OR = 0.77, 95% CI (0.48, 1.75)] at 95%CI with crude analysis*. Excess in mortality was observed among children with fathers' age 15-29 years when compared with groups 30-49 years.. Maternal age and paternal education were not significantly related to child survival. Higher level of wealth index was associated to a significant reduction in child mortality (see table 2). Excess in mortality was observed in children who reside in household with a floor made of earth than in a household with a floor made of cement. [OR = 1.93, 95%CI (1.002, 3.71).

**Table 2:** Factors associated with child survival, Jimma town, South Western Ethiopia October-November 2004

*Variable*	*Category*	*Cases*	*Controls*	*Crude OR (95% CI)*	Adjusted OR(95%CI)
Parity	1	22	80	1.00	1.00
	2-4	39	125	1.14(0.63, 2.05)	1.36(0.70,2.65)
	5 and above	12	16	2.73(1.13, 6.60)	3.34(1.27,8. 76)
Ever vaccinated	yes	53	206	0.14(0.07,0.32)	0.26(0.11,0.61)
	no	21	12	1	1
Ever breast fed	Yes	59	219	1.00	1.00
	No	15	3	18.56(5.2,66.23)	13.21(3.28,53.16)
Wealth index	Poor	63	164	1.00	1.00
	Satisfied basic needs	11	58	0.54 (0.31,0.93)	0.54(0.29, 0.99)

variables included in the adjusted model are ethnicity, religion, fathers age, floor of the house hold, parity, ever breast fed, vaccination status and wealth index

Breast milk was substituted for 80 (27%) of children at different age. Among these children 73.8% stop breast feeding before or at age of 6 months. The median age of initiation of supplementary feeding was 4.0 months. The mean age at which supplementation began showed significant difference for survival. (4.83 vs. 4.20, F = 5.22, p = 0.023). Children never breast fed had higher risk of dying than those who were breast fed in their life time. This was found to be significant even after adjustment (*table *2).

Caretakers disclosed that 263 (88.9%) children had been vaccinated at least once. Excess in mortality was observed among children who had never been immunized, which was statistically significant in both the crude and adjusted analysis (table2).

The odd of death for mothers of children with parity of 5 or more was 2.73 times higher than parity of one. *(OR = 2.73 95%CI (1.13, 6.60))*, and the effect was maintained after adjustment (table 2).

Pneumonia was the largest immediate cause of death for 22 children (29.7%, 95% CI (19.66, 41.48)) followed by acute diarrhea and malaria contributing to 23%" 95%CI (13.99, 34.21)" of deaths each. Malnutrition contributed to 28(37.8%) of all deaths. (Table 3). HIV/AIDS was found to be contributing for 2(2.8%) 95% CI (0.33, 9.42) of deaths using the pediatric case definition. (Table 3)

**Table 3:** Proportionate mortality by of cause of death of under five children, Jimma town, South Western Ethiopia October-November 2004

*Diagnosis*	*Number*	*%*	*95% CI*
**Malnutrition**	28	37.8	(26.81,49.86)
**Pneumonia**	22	29.7	(19.66,41.48)
**Malaria**	17	23	(13.99,34.21)
**Acute Diarrhea**	17	23	(13.99,34.21)
**Meningitis**	5	6.7	(2.23,15.06)
**Measles**	5	6.7	(2.23,15.06)
**HIV/AIDS**	2	2.8	(0.33,9.42)
**Persistent diarrhea**	2	2.7	(0.33,9.42)
**Dysentery**	2	2.7	(0.33,9.42)
**Congenital malformation**	2	2.7	(0.33,9.42)
**Neonatal tetanus**	1	1.35	(0.31,7.30)
**Total**			

The sum may exceed the total number of deaths (the tool used is assumed to identify probable and other underlying causes of deaths)

Percentage calculated from total deaths

## Discussion

Among the major effective child survival interventions, breast feeding and immunization were found to be major determinants of child survival. Pneumonia contributed to the largest deaths in the study population followed by acute diarrhea and malaria.

We used an unsatisfied basic need assessment in this study. This composite scale was used for the following reasons: (I) Income measurements are rarely reliable measures of poverty in low income countries. (ii) Furthermore this measure also excludes other elements of deprivation such as housing, clothing, education and health care[19]This index is based on different dimensions: housing quality, dependency ratio, availability of water supply and latrine [19] The wealth score which is a modification of the unsatisfied basic need assessment modified for local use was found to be a significant predictor of child survival.

Contrary to studies elsewhere [22-24] maternal education was not significantly associated to child mortality. In some studies the effect of education [on mortality] was significant among families with poor socioeconomic status[25]. Taking this into consideration, the analysis by stratification with wealth index did not show any significant difference. This might be attributed to the similarities that exist in the level of education between the comparison groups. Among environmental factors, a floor made of earth was significantly related to an excess in mortality when compared with cement floor. Its effect was lost as adjusted by other factors.

Breast feeding was found to be a strong predictor of child survival in this study. Breast feeding protects of infants from enteric infections by elimination of exposure to food- and water-borne pathogens; also, mature breast milk contains several compounds which increase immunity [26].

Higher level of protection by breast feeding was found among less educated women. Infants who were not breast fed had highest risk of dying during the first 2 months of life[27]. Mean age of weaning among cases was at earlier age than that of controls. Earlier weaning was also related to excess in child mortality in other studies [1,28]

Among preventive health services sampled, children's vaccination status was a strong predictor of child survival. The risk of mortality was about 6 fold higher among those who were not vaccinated, compared with those who received vaccination. The protective effect of vaccine is discussed in literatures [29,30]. The national coverage of immunization in Ethiopia differs by antigen. The BCG coverage has reached 70%, while the rate of fully immunized children was 36% [31]. The utilization of immunization service indirectly indicates caretakers' use of preventive health services.

Having more than five children was associated with increased risk of losing babies; the significance was kept even after adjusted for potential confounding variables. Other reproductive factors were not found to be significantly related to child survival.

Higher parity, short birth interval and low birth weight are known to be interrelated. The growth and development of young children are affected by their mothers' past nutritional histories and their well being during pregnancy. Birth weight is affected by maternal nutrition and health. High parity and short birth interval are known to deplete maternal nutrition and influencing the child survival through birth weight [32,33]

Measurement of causes of death is needed for several purposes: (i) To establish the relative public health importance of the different causes of death; (ii) To evaluate trends over time especially as a method of evaluating the probable impact of the intervention programs; and (iii) To investigate the circumstance surrounding the deaths of children from specific causes and to devise effective actions to decrease mortality[34].

Pneumonia contributes to the largest mortality in the study population, followed by acute diarrhea and malaria contributing death similar to national report [31]. The sensitivity and specificity of verbal autopsy diagnosis for malaria, acute respiratory infection and meningitides is low because these diseases share similar symptoms[35]. Use of these results warrants caution. Measles, neonatal tetanus, malnutrition and accidents were detected by verbal autopsy with sensitivities and specificities of greater than 75%[36]. Malnutrition contributed for 37.8% of all deaths. The contribution could be underestimated due to lack of ability of the tool to identify mild and moderate malnutrition. Identification of mild to moderate malnutrition using verbal autopsy is important since these groups are implicated in many more child deaths than previously recognized[32]. Hospital based data in developing countries suggest that 55-75% of perinatal deaths are associated with prematurity and low birth weight[37]. Because of the majority of deaths occur at home, birth weight measures are seldom available. Estimates of gestational age are particularly problematic in societies where often women do not know the dates of their last menstrual period. Birth trauma or asphyxia due to complications of delivery/poor obstetrical care are also thought to be major problems. But there exist little information regarding births that take place outside hospitals [37]..

HIV/AIDS was found to have caused 2(2.8%) of deaths using WHO pediatric HIV/AIDS definition. In other studies the ability to use verbal autopsy to distinguish deaths associated with maternal HIV infection from death by other causes was poor[21] The estimated contribution of HIV to under five mortality in Ethiopia was 8.1% which was based on data from sentinel surveillance and considering the rate of mother to child transmission, and survival time of infected children [33]. The difference between these two estimates could be due to limited ability of verbal autopsy to identify HIV-related death[38].

In conducting verbal autopsy different considerations should be taken into account. This study used an open approach in classification of death, broader category, and checklist without filter. The respondents were care takers. The interviewers used were lay persons trained intensively to conduct the study, which is known to increase the repeatability of the diagnosis when compared to medically trained people [15]. Recall period advisable was for 12 months since the event is relatively more common than adult deaths. On the other hand, others argue that mothers are intimately involved in the care of sick children and so they may report the symptoms preceding the death of a child more accurately than a relative caring for an adult [15,39].

With regard to validation of the tool, the option which was feasible was hospital-based reference which has many limitations. Besides this, we used verbal autopsy to estimate major causes of death.

### Limitations of the study

Most case control studies, except for nested case control design, fail to delineate the temporal relationship of exposure and outcome variables. Recall bias might have been introduced as some events and exposures are difficult to be remembered over one year period.

To minimize selection bias all remote and accessible kebeles/units were included in the baseline survey. The definition for cases and controls were strictly adhered to during the data collection. Some cases could be missed because of maternal recall of events that occurred in early neonatal period.

Low level of sensitivity and specificity were reported for acute respiratory infections, malaria and meningitis.

Difficult to make comparison of health care utilization between the groups as controls might not have experienced an illness that is comparable to cases.

## Conclusion

Immunized children were less likely to die as compared to unimmunized children. This can be considered as a proxy measure to health service access. Children born to mothers with fewer parity were less likely to die as compared to with parity of 5 or more. Breast fed children were less likely to die, compared to those never breast fed.

Based on these conclusions, we recommend the strengthening of immunization, family planning and breast feeding services.

## Competing interests

The authors declare that they have no competing interests.

## Authors' contributions

BGB: involved from the inception to design, acquisition of data, analysis and interpretation of data; 2) has been involved in drafting the manuscript or revising it critically for important intellectual content; and 3) has given final approval of the version to be published. YB: involved from inception to design, and interpretation of data; 2) has been involved in drafting the manuscript or revising it critically for important intellectual content; and 3) has given final approval of the version to be published

## Pre-publication history

The pre-publication history for this paper can be accessed here:

http://www.biomedcentral.com/1471-2458/11/197/prepub

## Supplementary Material

Additional file 1 D**ata collection instrument used for the study.** Click here for file
