# Supplementary material for: Children who were vaccinated, breast fed and from low parity mothers live longer: A community based case-control study in Jimma, Ethiopia
Source: BMC Public Health. 2011 Mar 31;11:197. doi: 10.1186/1471-2458-11-197 (PMC3084172; doi:10.1186/1471-2458-11-197)
Supplement: Additional file 1 — Data collection instrument used for the study. [file 1471-2458-11-197-S1.DOCX]

**Instruction**

*This study is intended to identify the determinants and causes of under five mortality .We kindly request your participation in this study. As stated in the consent form, we would like to remind you that You have the right of not responding to questions and interrupt during the interviews .We would like to thank for your support.*

**Section 1: Background information on child and household**

**IDENTIFICATION FORM**

| **1.1 HIGHER** | **1.4 SERIAL NUMBER** |
| --- | --- |
| **1.2 KEBELE** | **1.5 NAME OF THE CHILD** _____________________ |
| **1.3 HOUSE NUMBER** | **1.6 IS THE CHILD ALIVE1.ALUVE**  **2DEAD** |
| **1.7SEX 1MALE**  **2.FEMALE** | |

| **Section 2. Information about care taker** | **Section 3**  **Socio demographic** |  |
| --- | --- | --- |
| 2.1 Name of care taker  _____________________________ | 3.1 Date of birth of child:  ___/___/___  dd mm yy | 3.5 Religion  1.Orthodox  2. Protestant  3. Muslim  4. Others specify(---------- |
|  | 3.2 Date of death (if not alive)  ___/___/___  dd mm yy | 3.6 income  _____________ |
| 2.2 Relationship to the child  1. father  2. mother  3. grand father  4. grand mother  5. other (specify)______ | 3.3 age of child __________  (write in months )  if neonatal death in days (specify ) | - 1. Is there a TV?   1.YES  2.NO |
|  | 3.4 ethnicity  1.oromo  2. Amhara  3. Tigre  4. Gurage  5.others (specify | 3.8 IS THERE A RADIO?  1. YES  2. NO |
|  |  | |

| **Section 4.Parental factors** | | | |
| --- | --- | --- | --- |
| 4.1 **paternal factors** | | **4.2 maternal factors** |  |
| 4.1.1 status | 1.alive 2.dead 9.not known | 4.1.1 status | 1.alive 2.dead 9.not known |
| 4.1.2 age | _______ | 4.1.2 age | _______ |
| 4.1..3 marital status | 1. married 2. single 3. divorced 4. widowed 5. others(specify | 4.1..3 marital status | 1. married 2. single 3. divorced 4. widowed 5. others(specify |
| 4.1.4 level of education | _________________ | 4.1.4 level of education | _________________ |
| 4.1.5 occupation | __________________ | 4.1.5 occupation | __________________ |

| **Section 5Reproductive factor** | |
| --- | --- |
| 5.1 age at first pregnancy **______** | 5.9 Ever use of Family planning methods?  1.yes 2.no 9.dont know |
|  | 510 Have you ever attended ANC clinic?  1.yes 2.no 9.dont kknow |
| 5.2 age at birth this child **_____** | 5.11how many times?  _________________ |
| 5.3 parity **______** | 5.12Where did you deliver this child?   1. At home 2. Health institution 3. Others(specify |
| 5,4 Abortion _________ | 5.13Who attended it?   - 1. physician  1. nurse 2. Trained birth attendant 3. Lay person 4. Other (specify |
| 5.5birth interval between the selected child and the preceding child **______** |  |
| 5.6 birth interval between the selected child and next child  **______** |  |
| 5.7birth order  **______** |  |
| 5.8 any death in the household (of a child)  1.yes 2.no 9.dont kknow |  |

| **Section6 Environmental factors** | | | |
| --- | --- | --- | --- |
| **6.1Housing** | | **Sanitation**.6.2 | |
| 6.1.1. Type of roof | 1.thatched 2.corrugated iron 3.Made of brick 4.others specify | 6.2.1Source of water | 1.spring (protected) 2..river (zoned and protected3.spring 4.river 5.tap 6.others (specify ) |
| 6.1.2. Type of wall | l.1mud 2.cement 3.Iron 4.others(specify) | 6.2.2 Amount of water consumption | **_________-** |
| 6.1.3. Type of floor_____________ |  | 6.2.3 Is there a latrine facility | 1.yes 2.no 3.not known |
| 6.1.4. Number of rooms in the household_ | ____________ | 6.2.4Do you wash hands before feeding your child? | 1. Yes 2.no 9.dont know |
| 6.1.5.Number of windows in the household | ____________ | 6.2.5 Do you use soap when washing your hands? | 1. Yes 2.no 9.dont know |
| 6.1.6. Family size | ____________ |  |  |
| 6.1.7Do you cook in the household? | 1. Yes 2.no 9.dont know |  |  |
| 6.1.8 What kind of cooking material do you use ? |  |  |  |

| **Section 7**  **Nutritional factors** | | **Section 8**  **Child factor** | |
| --- | --- | --- | --- |
| 7.1Have you ever breast fed your index child? | 1. Yes 2.no 9.dont know. | - 1. What was the size of the child at birth? | 1.very small  2.smaller than usual  3.average  4. large |
| **7.2.** Are you currently breast feeding your child? (If the child is alive | 1. Yes 2.no 9.dont know. | - 1. was s/he immunized ? | Yes 2.no 9.dont know |
| 7.3 if the child is dead were you feeding the child just before he died?) | 1. Yes 2.no 9.dont know. | 8.3 is the card available ? | Yes 2.no 9.dont know |
| 7.4. If your child is >4-6 months, was s/ he exclusively fed? | 1. Yes 2.no 9.dont know. | 8.4 check for BCG,DPT1,DPT2,DPT3,OPV1,OPV2,OPV3 &measles | Circle on antigen received |
| 7.5 Have you ever substituted breast milk? | 1. Yes 2.no 9.dont know. | **8.5 If no card available ?**  8.5.1 Look for BCG scar / | Yes 2.no |
| 7.6 age breast milk substituted |  | 8.5.2 Was s/he ever vaccinated on the thigh or buttock ? | Yes 2.no 9.dont know |
| 7.7 have you ever given complimentary foods? | 1. Yes 2.no 9.dont know. | 8.5.2.1How many such vaccines? | _________________ |
| 7.8 age additional food started |  | 8.5.3 Did your child receive drops in the mouth? | Yes 2.no 9.dont know |
| 7.9. any bottle feeding ? | 1. Yes 2.no 9.dont know. | 8.5.3.1 When was it started ? | _________________ |
| 7.10 Problem of vision during night time | 1. Yes 2.no 9.dont know. | 8.5.3.2 How many times was it given? | _________________ |
| 7.11 Problem of vision during dayt time | 1. Yes 2.no 9.dont know. |  |  |
| 7.11 Problem of vision during day and night time  1. Yes 2.no 9.dont know. | | 1. Yes 2.no 9.dont know. | Yes 2.no 9.dont know |
|  |  |  |  |

| **Section 9**  ***Behavioral factors*** | | |
| --- | --- | --- |
| **9.1 Sub section I (PRACTICE )** | |  |
| 9.1.1Give additional food and fluid when child gets ill. | 1. Yes 2.no 9.dont know |  |
| 9.1.2 Give ORS to a child when having diarrhea | 1. Yes 2.no 9.dont know |  |
| 9.1.3 Do you take your child to health facility during illness? | 1. Yes 2.no 9.dont know |  |
| 9.1.4 Do you use ITN to your child for prevention of malaria? | 1. Yes 2.no 9.dont know |  |

= .

| **Section 9 contin…** | | | |
| --- | --- | --- | --- |
| **9.2 sub section 2(perception**  linker score will be used  1.strongly agree ,2agree 3. no comment 4.disagree 5.strongly disagreeree | | **93 subsection 3 (knowledge )** | |
| **9.2.1perceived susceptibly** |  | 9.3.1treatment is available for pneumonia at health institution | 1. Yes 2.no 9.dont know |
| 9.2.1.1child gets easily diarrhoea | 1__.2__.3__.4.__5__ |  |  |
| 9.2.1.2child gets easily ARI if not immunized | 1__.2__.3__.4.__5__ | 9.3.2Diarrhoal disease can be prevented by exclusive breast feeding. | 1. Yes 2.no 9.dont know |
| 9.2.1.3child can get and easily be affected by malaria if not protected and treated | 1__.2__.3__.4.__5__ |  |  |
| 9.2.1.4child get malnourished if not breast fed | 1__.2__.3__.4.__5__ | 9.3.3diarrhoea in children can be prevented by washing hands using soup | 1. Yes 2.no 9.dont know |
| **9.2.2 perceived severity**  **9.2.2.1**diarrhoea kills children | 1__.2__.3__.4.__5__ |  |  |
| 9.2.2.2ARI kills children | 1__.2__.3__.4.__5__ | 9.3.4sunshine exposure prevents deformity of the bones | 1. Yes 2.no 9.dont know |
| 9.2.2.3Malaria is a killer disease | 1__.2__.3__.4.__5__ |  |  |
| 9.2.2.4Malnutrition is major factor contributed children deaths | 1__.2__.3__.4.__5__ | 9.3.5When a child is having diarrhoea it is better to limit fluid to minimize loss | 1. Yes 2.no 9.dont know |
| **9.2.3perceived benefits**   - - - 1. ORS saves lives | 1__.2__.3__.4.__5__ |  |  |
| 9.2.3.2Additional food and fluid benefits a child with diarrhoea | 1__.2__.3__.4.__5__ | 9.3.6ITN prevents malaria transmission | 1. Yes 2.no 9.dont know |
| 9.2.3.3Sunshine exposure protects a child from getting bone deforimity | 1__.2__.3__.4.__5__ |  |  |
| 9.2.3.4ITN prevents chidren from getting malaria | 1__.2__.3__.4.__5__ | 9.3.1Maternal to child transmission of HIV is inevitable. | 1. Yes 2.no 9.dont know |
| 9.2.3.5Earlytreatmentpreventscomplication and deaths due to malaria pneumonia ,and others’ immunization prevents vaccine preventable deaths among children | 1__.2__.3__.4.__5__ |  |  |
| **9.2.4perceived barriers**  9.2.4.1ORS is expensive | 1__.2__.3__.4.__5__ | 9.3.7Measles and polio can be prevented by immunization | 1. Yes 2.no 9.dont know |
| 9.2.4.2ORS is not available at drug vendor ,pharmacy and clinic | 1__.2__.3__.4.__5__ |  |  |
| 9.2.4.3Health service too far | 1__.2__.3__.4.__5__ |  | |
| 9.2.4.4Health provider judgemental ,not friendly | 1__.2__.3__.4.__5__ |  |  |
| 9.2.4.5Side effect of drugs | 1__.2__.3__.4.__5__ |  |  |
| 9.2.4.6Transport to costly | 1__.2__.3__.4.__5__ |  |  |

**PART II**

**This part is applicable only for controls**

**Serious morbidity: defined as any single condition fulfilling the following criteria.**

- Shortness of breath
- Abnormal body movement
- Limitation of movement
- Lethargic
- Lost consciousness
- Unable to drink or eat

|  | **questions** |  |
| --- | --- | --- |
| 1. | Did your child have serious morbidity in the last one year? | 1. Yes 2.no 9.dont know |
| 2. | If yes, was s/he taken to health facility? | 1. Yes 2.no 9.dont know |
| 3. | If no to question2, what were the reasons for it? | 1.sought help from traditional healers  2.tried to treat child at home  3.transport not available  4.lack of recognition of transport  5.lack of confidence on medical care  6.other (specify)__________________ |
|  | **Questions 4,5,and 6 are for those who went to health facilities** |  |
| 4. | Was there a problem in getting appointment? | 1. Yes 2.no 9.dont know |
| 5. | was treatment available locally / | 1. Yes 2.no 9.dont know |
| 6 | .was there emergency care after consultation hours / | 1. Yes 2.no 9.dont know |
| 7 | .was there needed medical care? |  |

**Part III verbal autopsy form for neonate1111**

| 11 Symptoms mothers | | | | |
| --- | --- | --- | --- | --- |
| 1 | | How is the mother now? (1-healthy; 2-sick; 3-dead; 9-don’t know) | | 1 - 2 - 3 - 9 |
| 2 | | Did the mother have blood pressure? | | 1-Y 2-N 9-D |
| 3 | | Did the mother have fits before giving birth? | | 1-Y 2-N 9-D |
| 4 | | Did the mother have any of the following diseases: 1-diabetes, 2-heart diseases, 3-TB, 4-epilepsy? 5-No, 9-don’t know. | | 123459 |
| 5 | | Was it a difficult birth? | | 1-Y 2-N 9-D |
| 6 | | Did the mother have a febrile illness at the time of delivery? | | 1-Y 2-N 9-D |
|  | |  | |  |
| EVENTS DURING BIRTH OF A CHILD | | | | |
| 7 | | Was the child a single birth (1) or twin (2)? | 1- 2- 9-D | |
| 8 | | Was it a forceps or vacuum delivery? | 1-Y 2-N 9-D | |
| 9 | | Was it a caesarian delivery? | 1-Y 2-N 9-D | |
| 10 | | Child delivered feet first? | 1-Y 2-N 9-D | |
| 11 | | Excessive bleeding | 1-Y 2-N 9-D | |
| 12 | | Was it a prolonged labour? | 1-Y 2-N 9-D | |
| 13 | | Was the child premature? | 1-Y 2-N 9-D | |
| 14F | | How many months or weeks? | _____m\|_____w | |
| 15 | | Did water break before labor? | 1-Y 2-N 9-D | |
| 16F | | How much before labor did the water break? | 1.less than one day  2. one day or more | |
| 17 | | Did the baby stop ‘playing’ in the womb before labour? | 1-Y 2-N 9-D | |
| 18 | | If no, did the baby breathe at all after delivery? | 1-Y 2-N 9-D | |
| 19 | | If no, was the baby dead when s/he was born? | 1-Y 2-N 9-D | |
| 20 | | Did the umbilical cord come before the baby was born? | 1-Y 2-N 9-D | |
|  | |  |  | |
| ASK THESE QUESTIONS IF THE CHILD WAS BORN ALIVE | | | | |
| 21 | Did the child cry immediately after birth? | | 1-Y 2-N 9-D | |
| 22 | Was the child unable to breast-feed? | | 1-Y 2-N 9-D | |
| 23 | If yes, was the problem with the child (1) or the mother (2)? | | 1- 2- 9-D | |
| 24 | Was the child weighed after being born? | | 1-Y 2-N 9-D | |
| 25 | If yes, how much did the child weigh? | | __________Kgs | |
| 26 | At the time of birth was the newborn? | | 1.very small  2.smaller than usual  3.average  4. large | |
| 27 | Did the child have any malformation at birth? | | 1-Y 2-N 9-D | |
| 28 | Did the eye colour change to yellow (jaundice)? | | 1-Y 2-N 9-D | |
| 29 | If yes, how many days after being born? | | Days_____ | |
| 30 | Was the umbilical cord of the child RED colour? | | 1-Y 2-N 9-D | |
| 31 | Did the child have fever? | | Days_____ | |
| 32 | Did the child have convulsions? | | Days_____ | |
| 33 | Did the child become unconscious? | | Days_____ | |
| 34 | Was the child coughing? | | Days_____ | |
| 35 | Did the child have difficult in breathing? | | Days_____ | |
| 36 | Ever stop breathing? | | Days_____ | |
| 37 | Did s/he have fast breathing? | | Days_____ | |
| 38 | Did s/he have noisy breathing? | | Days _____ | |
| 39 | Did s/he have indrawing of the chest while breathing? | | Days_____ | |
| 40 | Did the child vomit? | | Days_____ | |
| 41 | Did the child have diarrhoea? | | Days_____ | |
| 42 | Was the child unable to breast-feed when s/he was ill? | | Days_____ | |
| 43 | Was there a bulge in the child’s fontanel? | | Days_____ | |
| 44 | Stiffness of the body ? | | Days ____ | |

**Part iV Verbal autopsy form: 31 days upto 59 months**

| Symptoms | | Months | Days |  |
| --- | --- | --- | --- | --- |
| 1 | Was the child too small at birth? | 1-Y 2-N 9-D | | |
| 2 | Was the child born premature? | 1-Y 2-N 9-D | | |
| 3-F | If yes, how many weeks or months? | _____m\|_____w | | |
| 4 | Was the child breast-feeding? | 1-Y 2-N 9-D | | |
| 5-F | If yes, did the child stop just before death? | 1-Y 2-N 9-D | | |
| 6 | Did s/he have fever? | _______\|______ | | |
| 7-F | Was the fever continuous (1) or on and  off (2)? | 1- 2- 9-D | | |
| 8-F | Did s/he have convulsions? | _______\|______ | | |
| 9 | Did s/he have a cough? |  | | |
| 10 | Repeated cough in last one year | 1.yes  2. noi  3.DK | | |
| 11 | How frequent? |  | | |
| 12 | Did s/he have breathing difficulties? | _______\|______ | | |
| 13 | Did s/he have fast breathing? | _______\|______ | | |
| 14 | Did s/he have noisy breathing? |  | | |
| 15 | Did s/he have indrawing of chest while breathing? | _______\|______ | | |
| 16 | Did S/he vomit? | _______\|______ | | |
| 17-F | Did S/he vomit blood? | _______\|______ | | |
| 18 | Did she have a mass in the abdomen? | _______\|______ | | |
| 19 | Did s/he have abdominal distension? | _______\|______ | | |
| 20-F | Did the distension start suddenly within few days (S) or gradually as the weeks went by (G)? | 1-S 2-G 9-D | | |
| 21 | Did s/he have diarrhoea? | _______\|______ | | |
| 22r | Repeated diarrhoea in the last one year? | 1.yes  2. noi  3.DK | | |
| 23 | How frequent? |  | | |
| 24 | Did s/he have bloody diarrhoea? | _______\|______ | | |
| 25 | Did s/he have abdominal pain? | _______\|______ | | |
| 26 | Did s/he have weight loss? | _______\|______ | | |
| 27 | Did s/he have flake of patches? | _______\|______ | | |
| 28 | Did s/he have hair changes in color?? | _______\|______ | | |
| 29 | Did s/he have mouth sores? | _______\|______ | | |
| 30 | Did s/he look pale? | _______\|______ | | |
| 31 | Did she have puffiness of the face? | _______\|______ | | |
| 32 | Did s/he have swollen legs |  | | |
| 33 | Did the eye colour change to yellow (jaundice)? | _______\|______ | | |
| 34 | Did she have ankle swelling? | _______\|______ | | |
| 35 | Did s/he have swelling of joints? | _______\|______ | | |
| 36 | Did she have skin rash? |  | | |
| 37 | Did s/he have measles? | _______\|______ | | |
| 38 | Did s/he have any other skin disease? | _______\|______ | | |
| 39 | Was s/he unusually sleepy? | _______\|______ | | |
| 40 | Did s/he have loss of consciousness? | _______\|______ | | |
| 41 | Did s/he have paralysis of both legs? | _______\|______ | | |
| 42 | Did s/he develop stiffness of the whole body? | _______\|______ | | |
| 43 | Did s/he have neck pain? | _______\|______ | | |
| 44 | Did s/he have fits? | _______\|______ | | |
| 45 | Did s/he have headache? | _______\|______ | | |
| 46 | Did s/he have bulging fontanelle? |  | | |
| 47 | Was s/he unable to pass urine? | _______\|______ | | |
| 48 | Did s/he pass blood in urine? | _______\|______ | | |
| 49 | Did s/he have swelling in the armpits? |  | | |
| 50 | Did s/he have swelling over the groin? |  | | |
| 51 | Did a dog bite him/her? | _______\|______ | | |
| 52 | Was s/he bitten by another animal or insect? | _______\|______ | | |
| 53 | If yes, what type of animal/insect? (mention the name) | _____________ | | |
| 54 | Was s/he injured in a road accident? | _______\|______ | | |
| 55 | Did s/he suffer any other accidental injuries? | _______\|______ | | |
| 56 | Was s/he injured intentionally by someone? | _______\|______ | | |

| Serial no | | questions |  |
| --- | --- | --- | --- |
| 44.1 | | Was the child taken to health facility in the last illness? | 1. Yes 2.no 9.dont know |
| 44.2. | | If yes to question no 1.where / | 1hospital  2. health center  3.health station  4.private clinic  5.others ____________ |
| 44.3. | | If no to question1, what were the reasons for it? | 1.sought help from traditional healers  2.tried to treat child at home  3.transport not available  4.lack of recognition of transport  5.lack of confidence on medical care  6.other (specify)__________________ |
| **Questions 4,5,and 6 are for those who went to health facilities** | | | |
| 44.4 | | Was there a problem in getting appointment? | 1. Yes 2.no 9.dont know |
| 44.,5. | | was treatment available locally / | 1. Yes 2.no 9.dont know |
| 44.6 | | .was there emergency care after consultation hours / | 1. Yes 2.no 9.dont know |
| 44.7 | | .was there needed medical care? | 1. Yes 2.no 9.dont know |
| 57.Cause of death mothers percepation | |  |  |
| 58. place of death | | 1.home  2.hospital  3. health center  4.other specify |  |
